# Radicalization and violent extremism depend on envy; conspiracy ideation, sometimes

**DOI:** 10.3389/fpsyg.2023.1111354

**Published:** 2023-03-29

**Authors:** Michael Moncrieff, Pierre Lienard

**Affiliations:** ^1^Department of International Public Law and International Organization, University of Geneva, Geneva, Switzerland; ^2^Geneva Academy of International Humanitarian Law and Human Rights, Geneva, Switzerland; ^3^Department of Anthropology, University of Nevada, Las Vegas, NV, United States

**Keywords:** envy, radicalization, extremism, violence, conspiracy ideation

## Abstract

Emotions are conspicuous components of radicalization, violent extremism, and conspiracy ideation. Of the emotions studied for their contribution to those social pathologies, envy has been relatively unexplored. We investigate the relationship between envy, radicalization, and conspiracy ideation. Envy appears to affect core aspects of radicalization, particularly the endorsement of extremism and the acceptance of violent means to achieve one’s ends, while radicalization facilitates the adoption of conspiracy ideation, rather than the latter being a cause of radicalization. Implications for future research on radicalization and violent extremism are discussed.

## 1. Introduction

Radicalization identifies the process by which a person comes to adopt extreme political, social, or religious ideologies and attitudes, often to the point of supporting or engaging in acts of violence (see Borum, 2011; Hafez and Mullins, 2015). Conspiracy ideation involves the perception that groups of social agents might be colluding to achieve nefarious objectives (Imhoff et al., 2022). Political radicalization, violent extremism, and conspiracy ideation are often lumped into the selfsame family of phenomena. For instance, that conspiracy beliefs would be at the source of extremism and violence is repeatedly advanced in mass media (Kunzelman, 2022) and within academic circles (e.g., van Prooijen et al., 2015; Jolley et al., 2022). Might these distinct phenomena actually partake of shared psychologies? Both conspiracy ideation and radicalization involve the adoption of extreme beliefs and ideologies and a similar sense of paranoia and/or distrust of the established order (e.g., van Prooijen et al., 2015; Vegetti and Littvay, 2022). Anxiety, humiliation, shame, guilt, contempt, pride, and elation, among other emotions, have been linked to the above-mentioned social pathologies (e.g., Cottee and Hayward, 2011; Tausch et al., 2011; van Stekelenburg, 2017; Wolfowicz et al., 2020; Cottee, 2021). However, the contribution of envy, in effect, has been left unexplored (but for some discussions see, Meloy et al., 2004; Vetlesen, 2006; Fiske, 2011; Knoll and Reid Meloy, 2014; Myketiak, 2016; Trip et al., 2019).

Envy is a complex emotion motivating the agent to track the advantages others are perceived to have, to be sensitized to the purported fitness-suppressing consequences those might have for the envious individual, and to attend to the eradication of the differential (e.g., Schoeck, 1966; Smith and Kim, 2007; Fiske, 2011; Sznycer et al., 2017, 8421).^1^ We propose that the functional structure of envy may serve as the mediator to subsequent downstream emotional and cognitive states studied in relation to the phenomenon of radicalization, violent extremism, and, potentially, conspiracy ideation. Why? The motivational feature of envy promotes the monitoring of potential welfare risks (Hill and Buss, 2008), which are revealed by proxy cues of social differentiation (van de Ven and Zeelenberg, 2020). A negative appraisal of one’s social condition, associated with frustration, uncertainty, and the desire to improve one’s situation, characterize the oft-cited catalyst of the radicalization process (e.g., Borum, 2003; Horgan, 2005; Moghaddam, 2005; Wiktorowicz, 2005; Silber et al., 2007; Sageman, 2008; Hafez and Mullins, 2015). We argue that the functional structure of envy constitutes the core element necessary, but not sufficient, for the process of radicalization to be set in motion. Furthermore, envy may also factor in the development of conspiracy beliefs, as an individual who would feel envious of others may attribute others’ success to collusion (see Winiewski et al., 2015).

An examination of envy’s functions (see, Hill and Buss, 2008; Cohen-Charash and Larson, 2016; Sznycer et al., 2017) reveals striking characteristics that may contribute to explaining essential and conspicuous features of the radicalization process: the motivation to monitor social differentials, the identification of the source of postulated welfare costs, the impulse to eliminate or depower competitors, and the derivation of pleasure at the misfortune of the envied.^2^ Indeed, several features of envy-motivated behaviors and attitudes are ostensibly observed in extreme, radicalized or conspiracy theory comportments:

1. Threatening social differentiation prompts envy (Hill and Buss, 2008), which is often pointed toward perceived social superiors (Schoeck, 1966; Smith and Kim, 2007; Fiske, 2011; Sznycer et al., 2017). A negative social appraisal likewise accelerates political radicalization or violent extremism (Borum, 2003; Moghaddam, 2005). Envy thrives in contexts of social zero-sumness, whence one’s gain is interpreted as another’s loss (Alicke and Zell, 2008; van de Ven and Zeelenberg, 2020). So does political radicalization (e.g., van den Bos, 2020). A zero-sum logic spurs conspiracists to detect evidence of antagonist collusions of successful nefarious agents (Imhoff and Lamberty, 2020) and conspiracy themes find their analogs in the stereotypes of envy-motivated prejudices (Winiewski et al., 2015; Smith and Hoogland, 2019).
2. Envy may be activated when the attribution of direct and immediate responsibility for purported wrong is impossible to make or ambiguous (Lienard and Moncrieff, n.d.). Envy is a proactive emotion not requiring others’ harmful actions to be activated; the sheer existence of other agents is enough. During the radicalization process, Moghaddam (2005) identified a stage – displacement of aggression – where a radicalized agent’s negative feelings are attributed to a perceived causal agent (e.g., person, group, nation) that is responsible for one’s grievances. It is important to note here that we argue that radicalization is not primarily reactive but proactive. The proactive nature of radicalization would account for the phenomenon of “composite violent extremism” where one’s negative state leads to a hatred of “EVERYONE AND EVERYTHING” [capitalization in the original source] instead of a clearly defined target of aggression and to a “mixed, unstable, or unclear ideology” (Gartenstein-Ross et al., 2022).^3^ Likewise, individuals vary in their overall inclination toward accepting conspiracy theories, which may even extend to holding beliefs in conspiracy theories that conflict with each other (Bruder et al., 2013; Imhoff et al., 2022). Radical, extreme, and conspiracy ideations often implicate the identification of agents despised for no other reason than their sheer existence (Ware, 2020). Both unwarranted attribution of agency and intentionality are coupled with a support for conspiracy theories (Imhoff and Bruder, 2014; Douglas et al., 2016).
3. Envy is associated with hostile, aggressive and spiteful behaviors (Zizzo and Oswald, 2001; Miceli and Castelfranchi, 2007; Smith and Kim, 2007; Wobker, 2015; Morgan et al., 2022). The hallmark of radicalization and violent extremism is targeted aggression; even when it involves elevated personal costs (e.g., Atran, 2003). Schadenfreude (Smith et al., 1996), the jubilation at the suffering of the envied, matches the pleasure that radicalized violent extremists and conspiracy theorists experience upon their targets’ misfortune (Saucier et al., 2009; Winiewski et al., 2015).

To the authors’ knowledge, the strong resemblance of envy-motivated behaviors and attitudes with radicalized, violent extremist and conspiracy-theorist mindsets and comportments has not yet been empirically examined. How might they be related? Moncrieff and Lienard (2021)^4^ argued that radicalized individuals volunteer rationalizations that are unlikely to reveal the etiology of the process of radicalization followed and that more fundamental causes, such as emotions, most probably precede radical and extremist belief formation. Thus, the progression from conspiracy to the display of radical comportments should not be granted any significant explanatory privilege in the process of radicalization. On the other hand, envy could indirectly facilitate the adoption of conspiracy beliefs *via* radicalization. Note too that this does not imply that radicalization or envy should necessarily precede conspiracy beliefs, as such beliefs may be held for other reasons (e.g., to coordinate with others; see, Mercier and Altay, 2022).

We examine the relationship between dispositional envy, central aspects of radicalization (i.e., extremist attitude and endorsement of violence), and conspiracy mentality. We make the following predictions:

*H_1_*: Dispositional envy positively correlates with extremist attitudes and endorsement of violence.

*H_2_*: Dispositional envy indirectly facilitates the adoption of conspiracy beliefs via radicalization.

## 2. Methods

The study adhered to the Swiss national ethical guidelines for research involving human participants. It is in accordance with the ethical standards of the institutional research committee and the 1964 Helsinki declaration. Institutional review boards are not mandatory for certain types of exempt research activities in the country where the investigator responsible for the present study is employed (Switzerland). All data and study materials are available online on the Open Science Framework.^5^ The study was preregistered prior to the start of data collection.^6^

### 2.1. Participants and procedures

Participants (*n* = 447) were recruited from the United States in the Fall of 2022 using the Connect online platform by CloudResearch^7^ and directed to the Qualtrics^8^ survey website to complete all survey responses. The sample included 229 men and 212 women. Six individuals who did not state their sex were excluded from the analyses. The average age was 42 (range = 19–84). On a 7-point political orientation scale measuring one’s liberal v. conservative political orientation, 1 (*very liberal*) to 7 (*very conservative*), with 4 (*moderate*) as mid-point, the mean response was 3.5 (SD = 1.81).^9^ After reading and agreeing to the informed consent, participants were presented with the Dispositional Envy Scale, Conspiracy Mentality Questionnaire, Extremism Scale, and the Pro-violence in Relation to Extremism Scale. The order of the survey measures and the order of the items on each survey were randomized. At the end of the study, participants responded to demographic questions regarding their gender, age, and left/right political orientation. The mean response time was 4 min. Participants were compensated $0.70.

### 2.2. Dispositional envy

The Dispositional Envy Scale (Smith et al., 1999) consists of eight items measuring individual differences in their tendency to feel envy, coded on a scale ranging from 1 (strongly disagree) through 5 (strongly agree). The scale aims to measure individual differences in proneness to envy and has been validated in a large number of studies (for a conceptual review of dispositional envy, see Lange et al., 2018). One’s proneness to envy should serve as an adequate proxy for envy and its relationship to the other measures in our study. Examples of items include “I feel envy every day,” and “It is so frustrating to see some people succeed so easily.” Cronbach’s *α* was 0.89 for the scale.

### 2.3. Conspiracy mentality

The Conspiracy Mentality Questionnaire (Bruder et al., 2013) consists of five items assessing generic beliefs in conspiracy theories, coded on a scale ranging from 0% (certainly not) through 100% (certain). Scores were converted to 0–10 for analysis. The scale measures one’s general susceptibility to explanations based on conspiracy theories. The scale’s reliability and convergent, discriminant, and predictive validity was demonstrated in a range of studies (Bruder et al., 2013). Examples of items include “I think that government agencies monitor all citizens” and “I think that many very important things happen in the world, which the public is never informed about.” Cronbach’s *α* was 0.87 for the scale.

### 2.4. Extremism and acceptance of violent means

Endorsement of extremism and acceptance of violent means were measured using items from the Extremism Scale and the Pro-violence in Relation to Extremism Scale (Ozer and Bertelsen, 2018). These two scales are positively and significantly correlated, reflecting their assessment of related aspects of radicalization. Reponses were coded on a scale ranging from 1 (strongly disagree) through 7 (strongly agree). Examples of items from the extremism scale include “Those groups in the society that do not support the good and correct life should be deprived of their rights” and from the pro-violence scale include “Using physical violence is the only thing that really works when it is a matter of … creating a new and better society.” The goal of these generic scales is to measure central aspects of violent radicalization through extremist attitude and endorsement of violence.^10^ These measures should help us to see if one’s proneness to experiencing envy is related to certain dispositions and propensities associated with radicalization. To reduce the survey length, three items with the highest factor loadings in Ozer and Bertelsen (2018) were used from the 14 item Extremism Scale and three items from the original six item Pro-violence scale. Cronbach’s *α* was 0.74 for the Extremism scale and 0.90 for the Pro-violence scale.

## 3. Results

As hypothesized (Hypothesis H_1_), the data showed small (*r* = 0.22) to moderate (*r* = 0.40) statistically significant positive correlations for envy with extremism, pro-violence, and conspiracy (see Table 1). Controlling for age, sex, and political orientation the partial correlations remained statistically significant for envy with extremism (*r* = 0.40, *p* < 0.001), pro-violence (*r* = 0.36, *p* < 0.001), and conspiracy (*r* = 0.23, *p* < 0.001).

**Table 1:** Descriptive statistics and correlations for study variables.

Variable	*M*	SD	1	2	3	4	5	6
1. Envy	2.01	0.87						
2. Extremism	2.43	1.26	0.40^***^					
3. Violence	1.68	1.10	0.36^***^	0.64^***^				
4. Conspiracy	5.53	2.27	0.22^***^	0.34^***^	0.23^***^			
5. Age	42.09	12.20	−0.25^***^	−0.26^***^	−0.28^***^	−0.16^***^		
6. Sex	0.48	0.50	0.07	−0.11^*^	−0.24^***^	0.02	0.12^*^	
7. Political	3.50	1.81	−0.13^***^	0.16^***^	0.22	0.26^***^	0.14^*^	0.06

^*^p < 0.05. ^***^p < 0.001. For sex 0 = male, 1 = female.

To examine the relationship between the variables we deviate slightly from the preregistration plan and use Structural Equation Modeling (SEM) instead of Multivariate Regression. While both analyses use general linear modeling, SEM offers several advantages. SEM is robust to measurement error, allows the modeling of latent variables, permits the simultaneous testing of relationships including direct and indirect effects, and the identification of best-fitting models (Nunkoo and Ramkissoon, 2012). We rely on Stata Statistical Software (Release 17; StataCorp LLC. 2021) to complete the analyses.

**Figure 1:**
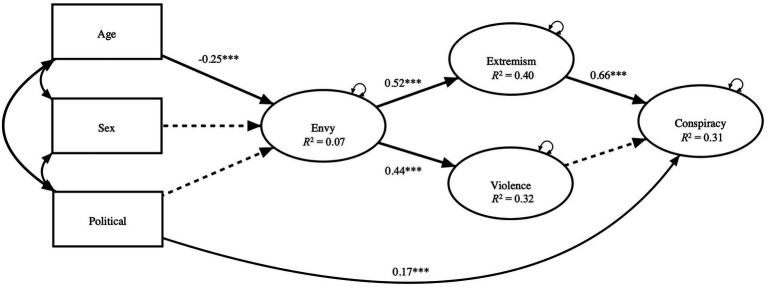
Structural path model showing standardized coefficients. ***p< 0.001. Some paths from demographics to the latent variables are omitted for clarity (see Table 2 for all paths). Dashed lines indicate non-significant paths.

In the SEM for testing our second hypothesis (H_2_), envy was located in an antecedent position to extremism and violence, the latter two preceding conspiracy (Figure 1). We suspected that common method bias might be an issue because of the potential for social desirability effects, particularly with the envy measure, and because we used the same participants for the collection of the independent and dependent variables. Harman’s single-factor test indicated that the variance accounted for by the common latent factor was within an acceptable range < 31% (Harman, 1960). To further reduce the plausibility of method biases as a rival explanation for the relationships observed in our study, we relied on the unmeasured latent method factor technique to conservatively correct the estimates in our model (Podsakoff et al., 2012, 553). The more conservative estimates did not significantly impact our interpretation of the model. Several items of the Dispositional Envy Scale and Conspiracy Mentality Questionnaire appeared to measure the same information and modification indices indicated a better model fit by allowing correlated residuals among these items (see Supplementary Information).

The data were non-normally distributed. Satorra–Bentler adjustments are robust to nonnormality and therefore used in the reporting of goodness of fit statistics. Based on the calculations of Stata SEM using maximum likelihood estimation, related indices of model fitness (with Satorra–Bentler adjustments) were: Normed Chi-square (*χ*^2^/DF) = 1.95, TLI = 0.945, CFI = 0.954, RMSEA = 0.047, 90% CI [0.46, 0.59], SRMR = 0.056. All the indices indicated that the research model yielded an acceptable fit.

The model indicated that envy had significant direct effects on extremism and violence (Table 2). The demographic variables accounted for only 7% of the variance in envy, while the combination of demographic variables and envy accounted for 40% of the variance in extremism and 32% of the variance in violence. When a path from envy to conspiracy was introduced into the model, envy had no statistically significant direct effect. However, envy had a significant indirect effect on conspiracy (*B* = 0.60, *p* < 0.001, 95% CI [0.37, 0.84]), supporting the second hypothesis (H_2_). Extremism had a positive direct effect on conspiracy in the model (*B* = 1.255, *p* < 0.001, 95% CI [0.75, 1.764]). Violence did not reach significance. Overall, the model accounted for 32% of the total variance in the observed variables.

**Table 2:** Table of all SEM direct and indirect path parameters.

Path	Parameters				95% conf. interval	
	*β*	*B*	SE	Value of *p*	LB	UB
Age						
→ Envy	−0.246	−0.017	0.003	< 0.001^***^	−0.022	−0.012
→ Extremism	−0.202	−0.018	0.004	< 0.001^***^	−0.027	−0.010
→ Violence	−0.159	−0.012	0.003	< 0.001^***^	−0.018	−0.007
IE → Conspiracy	−0.155	−0.027	0.005	< 0.001^***^	−0.037	−0.017
Sex						
→ Extremism	−0.098	−0.330	0.091	< 0.001^***^	−0.509	−0.151
→ Violence	−0.240	−0.504	0.080	< 0.001^***^	−0.661	−0.346
IE → Conspiracy	−0.008	−0.037	0.115	0.748	−0.263	0.189
Political						
→ Envy	−0.093	−0.043	0.024	0.069	−0.090	0.003
→ Extremism	0.269	0.164	0.033	< 0.001^***^	0.101	0.228
→ Violence	0.120	0.063	0.023	0.006^**^	0.018	0.107
→ Conspiracy	0.172	0.200	0.058	0.001^***^	0.085	0.315
Envy						
→ Extremism	0.522	0.684	0.097	< 0.001^***^	0.494	0.874
→ Violence	0.441	0.496	0.078	< 0.001^***^	0.342	0.649
IE → Conspiracy	0.241	0.602	0.119	< 0.001^***^	0.368	0.836
Extremism						
→ Conspiracy	0.658	1.255	0.260	< 0.001^***^	0.746	1.764
Violence						
→ Conspiracy	−0.232	−0.515	0.239	0.050	−1.024	−0.008

^**^p < 0.01. ^***^p < 0.001. IE, indirect effect; LB, lower bound; UB, upper bound. For sex 0 = male, 1 = female.

The strong direct effect between extremism and conspiracy supports the idea that the endorsement of extremism could prepare an individual for the acquisition of conspiracy beliefs. The direct effect of political orientation on conspiracy is also noteworthy. When controlling for the effects of envy, extremism, and violence on conspiracy in a partial correlation, age and sex are no longer statistically significant, however, political orientation is still significantly correlated with conspiracy (*r* = 0.251, *p* < 0.001). This finding suggests that an individual can hold conspiracy beliefs without the facilitating effects of envy or extremism attitudes, however, extremism may also play a prominent role for certain individuals in their adoption of conspiracy belief. We tested a range of competing models placing conspiracy before extremism, violence, and/or envy. We used AIC model selection to compare that set of plausible models to the model we have selected. In support of our second hypothesis (H_2_), our model has the best-fit (Table 3).

**Table 3:** AIC comparison of alternative models.

Model	Variable paths	𝐴𝐼𝐶*_m_* - 𝐴𝐼𝐶_min_
0^*^	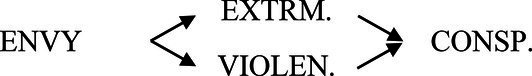	
1	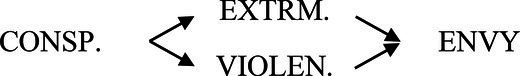	17.62
2	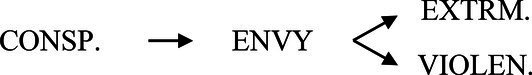	44.28
3	CONSP. → ENVY → EXTRM. → VIOLEN.	42.92
4	CONSP. → EXTRM. → ENVY → VIOLEN.	156.02
5	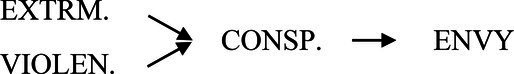	80.72
6	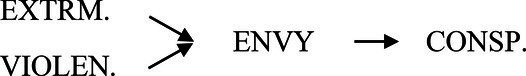	40.22
7	EXTRM. → VIOLEN. → CONSP. → ENVY	110.47
8	ENVY → EXTRM. → VIOLEN. → CONSP.	28.67
9	ENVY → VIOLEN. → EXTRM. → CONSP.	19.95
10	ENVY → EXTRM. → CONSP. → VIOLEN.	83.48

*AICmin = 32065.397. If AIC*m* - AICmin < 2, then there is substantial support for the alternative model. No models approached such thresholds.

## 4. Discussion

We find support for the hypothesis (H_1_) that envy has an impact on core aspects of radicalization, particularly the endorsement of extremism and acceptance of violent means. Few studies currently examine the relationship between envy, radicalization and violent extremism (but see, Moncrieff and Lienard, 2021). Our SEM suggests that investigating further this linkage might lead to novel insights. For instance, programs to ‘prevent’ or ‘counter’ violent extremism (P/CVE) may be more successful if they tackle envy as a core component of the process of radicalization. In line with our assumptions, a deradicalization intervention provider in the United Kingdom notes how his method involves “[persuading] clients *to take responsibility* [emphasis added] for their views or prejudices rather than blaming external factors” (Warrell, 2019). We also find support for the second hypothesis (H_2_) that conspiracy ideation can be related to envy, but indirectly, *via* the endorsement of extremism. Our model supports the position that a core aspect of radicalization may precede conspiracy beliefs in some circumstances, but be unrelated in other circumstances. Our research suggests that fundamental causes, such as emotions, are likely to be more important than explicit beliefs when it comes to engagement in costly behaviors. Further research will be required to tease apart the sequence of such events.

The obtrusive nature of some survey statements (e.g., “I feel envy everyday”) appears to pose a significant limitation. If revealed to others, envy comes with many social risks (Smith, 2004). Individuals may be unwilling to openly admit to having such feelings. The authors intend to conduct further studies using implicit measures of envy to mitigate potential response bias. Indeed, future studies should seek evidential diversity – using different methods and measures – to test the proposed model. Another limitation is the sample used in this study. Our sample was singly from the United States and did not target specific extremist communities. Future studies should examine the linkages between envy and radicalization in populations that are known to have a greater proportion of radicalized members (e.g., online extremist communities). Indeed, the effect of envy on radicalization may be significantly weaker in our general sample than in extremist social milieus. Future research should also examine the relationship between envy and its potential mediation effect on downstream emotions typically noted in the radicalization process (e.g., hatred, humiliation, shame, contempt, elation). Only with additional research will we know if envy is truly the core emotion constituting radicalization or if it is merely a related but noncausal aspect of the phenomenon.

## Data availability statement

The datasets presented in this study can be found in online repositories. The names of the repository/repositories and accession number(s) can be found at: https://osf.io/rjxsd.

## Ethics statement

Ethical review and approval was not required for the study on human participants in accordance with the local legislation and institutional requirements. The patients/participants provided their written informed consent to participate in this study.

## Author contributions

MM responsible for the design, data collection, statistical analyses, and writing of this paper. PL responsible for the design, interpretation of the findings, and writing of this paper. All authors contributed to the article and approved the submitted version.

## Funding

MM was supported by the Swiss National Science Foundation grant number 176781.

## Conflict of interest

The authors declare that the research was conducted in the absence of any commercial or financial relationships that could be construed as a potential conflict of interest.

## Publisher’s note

All claims expressed in this article are solely those of the authors and do not necessarily represent those of their affiliated organizations, or those of the publisher, the editors and the reviewers. Any product that may be evaluated in this article, or claim that may be made by its manufacturer, is not guaranteed or endorsed by the publisher.

## Footnotes

^1^The literature sometimes distinguishes between ‘benign envy’ and ‘malicious envy’ – in the former the agent competes whereas in the latter he aggresses. We conceptualize envy only as the malicious type which aligns with Cohen-Charash and Larson (2017) who argue that “envy, conceptualized and measured as a unitary construct, can lead to a wide range of reactions, both socially desirable and undesirable, depending on personal and situational moderators. We propose that the distinction between “benign” and “malicious” envy is unwarranted and advocate for the use of envy as a unitary construct.”

^2^While a full theoretical discussion is beyond the scope of this brief research report, see Lienard and Moncrieff (n.d.), for a more robust argument.

^3^Envy is triggered by a subjective evaluation not necessarily by objective circumstances (Smith and Kim, 2007). A good illustration of that fact is Duncan’s (2010) case study of a former German neo-Nazi leader who, despite an objective political reality suggesting otherwise, perceived himself and his fellow ethnic Germans as under attack and relatively deprived of political power.

^4^This article is currently posted as a preprint, which has been expanded and revised to be published in two articles. The first article is currently in press (Lienard and Moncrieff, 2023) and the second, which details our theoretical model about envy and radicalization, is in preparation.

^5^ https://osf.io/46fcj

^6^ https://aspredicted.org/85H_LYD

^7^The Connect platform on Cloudresearch.com monitors the quality of participants on their platform to enhance the collection of online research data. More information can be found on the company’s website.

^8^Qualtrics is a leading hosting platform for online surveys and experimental research (see Qualtrics.com for more information).

^9^While some debate exists regarding the most appropriate scale to measure political orientation (e.g., Kroh, 2007), scholars have been successful in measuring political orientation using a 7-point liberal/conservative scale in U.S. samples (see, Kim et al., 2012).

^10^Some types of radicalization (e.g., climate change radicalism) on the surface do not seem to feature the perception of a differential between haves and have-nots. However, these movements are still predicated on a zero-sum construal of the world (i.e., resources and power are finite, and any gains made can only be achieved at the expense of others) and the violent overthrowing of an existing status quo. It is not surprising that radicalized climate change activists favor economic de-growth as a means to address what they propose is an existential crisis. Indeed, zero-sum thinking and violent eradication of the perceived advantages that the status quo affords to some are also the hallmark of these types of radicalism.
