# Supplementary figures and images for: Radicalization and violent extremism depend on envy; conspiracy ideation, sometimes
Source: Front Psychol. 2023 Mar 29;14:1111354. doi: 10.3389/fpsyg.2023.1111354 (PMC10090496; doi:10.3389/fpsyg.2023.1111354)

**Supplementary Materials**

1. **Full model specification** (displaying standardized estimates)

**
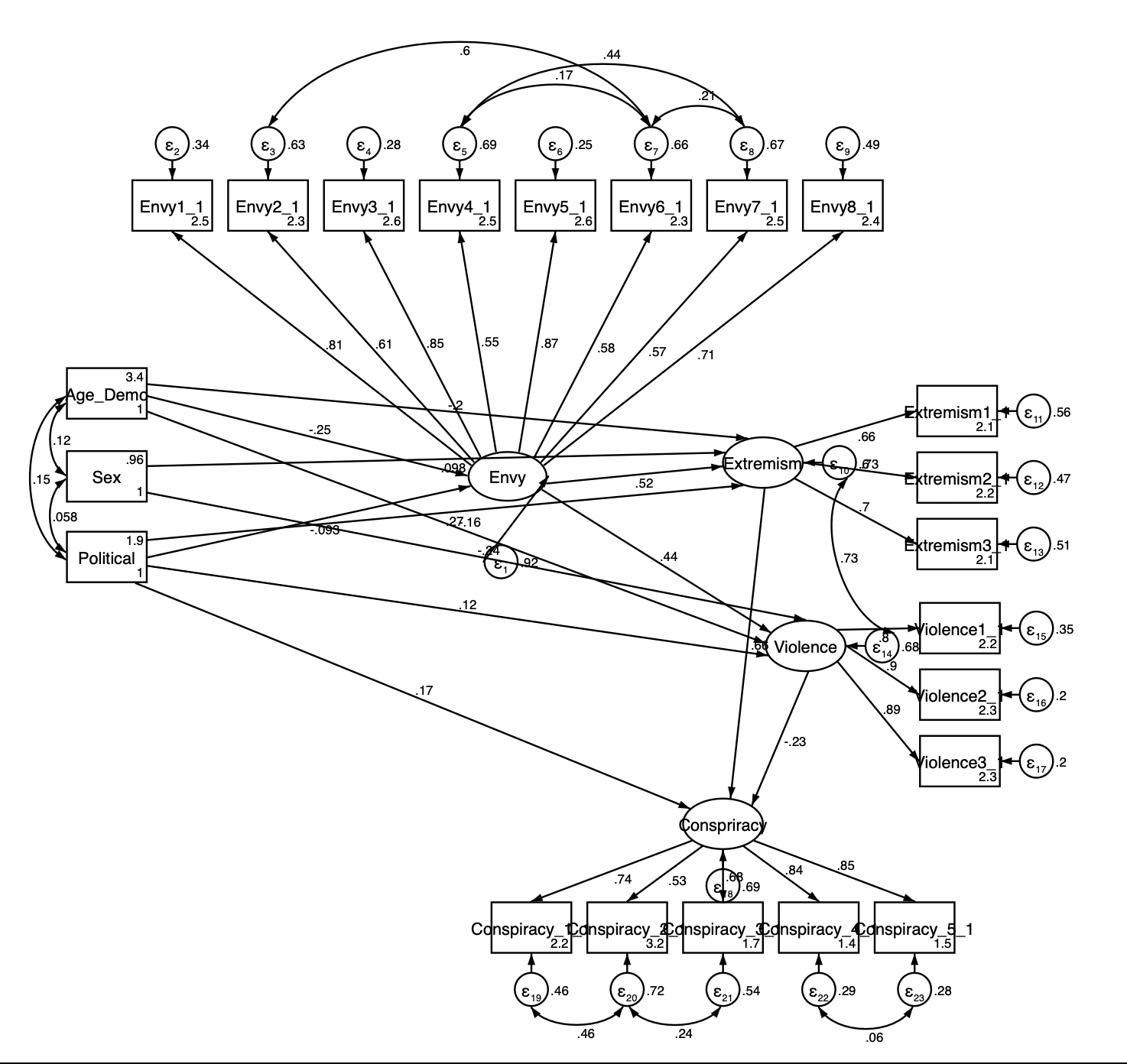
**

Supplement: Supplementary file 1 [file Data_Sheet_1.docx]
